# The obesity paradox in acute coronary syndrome: a meta-analysis

**DOI:** 10.1007/s10654-014-9961-9

**Published:** 2014-10-30

**Authors:** Jacek Niedziela, Bartosz Hudzik, Natalia Niedziela, Mariusz Gąsior, Marek Gierlotka, Jarosław Wasilewski, Krzysztof Myrda, Andrzej Lekston, Lech Poloński, Piotr Rozentryt

**Affiliations:** 1 Third Department of Cardiology, Silesian Center for Heart Diseases, Medical University of Silesia, M. Curie-Skłodowskiej 9, 41-800 Zabrze, Poland; 2 Department of Neurology, Medical University of Silesia, Zabrze, Poland

**Keywords:** Acute coronary syndrome, Obesity, Obesity paradox, Body mass index

## Abstract

In the general population, the lowest mortality risk is considered to be for the body mass index (BMI) range of 20–24.9 kg/m^2^. In chronic diseases (chronic kidney disease, chronic heart failure or chronic obstructive pulmonary disease) the best survival is observed in overweight or obese patients. Recently above-mentioned phenomenon, called obesity paradox, has been described in patients with coronary artery disease. Our aim was to analyze the relationship between BMI and total mortality in patients after acute coronary syndrome (ACS) in the context of obesity paradox. We searched scientific databases for studies describing relation in body mass index with mortality in patients with ACS. The study selection process was performed according to PRISMA statement. Crude mortality rates, odds ratio or risk ratio for all-cause mortality were extracted from articles and included into meta-analysis. 26 studies and 218,532 patients with ACS were included into meta-analysis. The highest risk of mortality was found in Low BMI patients—RR 1.47 (95 % CI 1.24–1.74). Overweight, obese and severely obese patients had lower mortality compared with those with normal BMI–RR 0.70 (95 % CI 0.64–0.76), RR 0.60, (95 % CI 0.53–0.68) and RR 0.70 (95 % CI 0.58–0.86), respectively. The obesity paradox in patients with ACS has been confirmed. Although it seems to be clear and quite obvious, outcomes should be interpreted with caution. It is remarkable that obese patients had more often diabetes mellitus and/or hypertension, but they were younger and had less bleeding complications, which could have influence on their survival.

**Electronic supplementary material:**

The online version of this article (doi:10.1007/s10654-014-9961-9) contains supplementary material, which is available to authorized users.

## Background

The concept of obesity (from the Latin word obdere—to eat all over: ob—over, above; edere—to eat) for the first time was used in the Oxford Dictionary in 1611, as a synonym for words: corpulent, thick [1]. The oldest trace of obesity is believed to be a female Willendorf statuette, dated about 22,000–24,000 years B.C. [2].

The attitude toward obesity has been changing over centuries and cultures. In ancient Greece (Hippocrates) and India (Sushruta), it was considered as a pathology [3]. In the Europe and the Far East, in the Middle Ages and the Renaissance, obesity was attractive and desirable. A corpulent silhouette was identified with wealth. In the twentieth and twenty-first century, obesity again became unpopular and unfashionable. Being slim has been considered as optimal weight status both for aesthetic and health reasons.

There are many parameters describing body weight status. Years of observation revealed that body mass and height were in certain proportions. Epidemiological significance of the same body weight is completely different in tall and short person. The most popular formula describing weight in relation to height is the Quetelet index, also known as Body Mass Index (BMI) [4]. BMI is expressed as the ratio of body weight in kilograms and the square of the height in meters. Based on epidemiological observations linking various aspects of health status with BMI, the World Health Organization (WHO) has established a normal BMI for European and North American populations in the range of 18.5–24.9 kg/m^2^ [5]. A BMI range of 25–29.9 kg/m^2^ defines overweight and a BMI of 30 kg/m^2^ and more is regarded as obesity. BMI below 18.5 kg/m^2^ indicates underweight.

In some populations, the BMI cut-off values for a diagnosis of obesity are different. For example, in the Japanese, South Korean and Chinese populations obesity is recognized for BMIs above 25 kg/m^2^ [6], 27.5 kg/m^2^ [7] and 28 kg/m^2^ [8], respectively.

BMI can be calculated easily and quickly and thus it is widely used both in research and clinical areas. It is also applied for body weight classification by WHO. It should be noted that BMI is not the only and probably not the most accurate measure of the cardiovascular risk associated with body weight.

The obesity, described as higher BMI, is considered as the risk factor for mortality in the general population. The lowest mortality is observed for the BMI range of 20–24.9 kg/m^2^ (for non-smokers in the American and European populations) and it increases below and above this range [5, 9]. During the last two decades, reports on the favorable prognosis in chronically ill patients with overweight or obesity have been published. This phenomenon commonly called the obesity paradox or reversed epidemiology was recognized in patients with chronic kidney disease [10], chronic heart failure [11] and chronic obstructive pulmonary disease [12]. Recently, a similar paradox linking higher BMI with better prognosis was described in coronary artery disease [13, 14]. Due to acute metabolic imbalance during AMI and increased catabolism following AMI [15], the occurrence of obesity paradox after AMI could be different than in stable CAD.

## Objectives

Our aim was to analyze the relationship between BMI and total mortality in patients after acute coronary syndrome (ACS).

## Methods

### Study design

The meta-analysis were performed according to the Preferred Reporting Items for Systematic Reviews and Meta-Analyses (PRISMA) statement [16].

### Data sources

PubMed, ScienceDirect and Cochrane Library databases were systematically searched for studies which reported total mortality rates in relation to BMI in patients with acute coronary syndrome. Multiple queries using following keywords were performed on August 27, 2014: (‘body mass index’ OR BMI OR ‘body weight’ OR obesity OR overweight OR underweight) AND (‘acute coronary syndrome’ OR ‘myocardial infarction’ OR ‘unstable angina’) AND (mortality OR death).

### Study eligibility criteria for qualitative and quantitative synthesis

Inclusion and exclusion criteria for qualitative and quantitative analyses were presented in Table 1. Studies fulfilling the eligibility criteria were included into analysis.

**Table 1:** PICOS criteria for inclusion and exclusion of studies into qualitative and quantitative (meta-analysis) analyses

Parameter	Inclusion criteria	Exclusion criteria
Qualitative synthesis criteria		
Patients	Adults with acute coronary syndrome (STEMI and/or NSTEMI and/or UA), regardless of treatment (MT, fibrinolysis, PCI, CABG) General population—studies with subgroups (i.e. age or sex) were included only if there was possibility to compile subgroups into one cohort	only Korean or Japanese population Population limited to a subgroup (i.e. age > 65 years old or men only included)
Intervention	Groups of BMI	Studies without BMI groups
Comparator	Normal BMI group	–
Outcomes	All-cause (total) mortality	–
Study design	Randomized controlled trials Non-randomized controlled trials Retrospective, prospective, or concurrent cohort studies Cross sectional studies	Case reports Editorials & opinion pieces
Quantitative synthesis criteria^a^		
Patients	–	–
Intervention	Low BMI, overweight, obesity, severe obesity (at least one of them)	No BMI groups
Comparator	Normal BMI group	No possibility to extract normal BMI group
Outcomes	All-cause (total) mortality expressed as mortlaity ratio, odds ratio or risk ratio	Lack of mortality defined in BMI groups
Study design	–	–

^a^Quantitative synhesis criteria contain criteria for qualitative synthesis

*PICOS* patients, intervention, comparator, outcomes, study design; *ACS* acute coronary syndrome; *BMI* body mass index

**Fig. 1:**
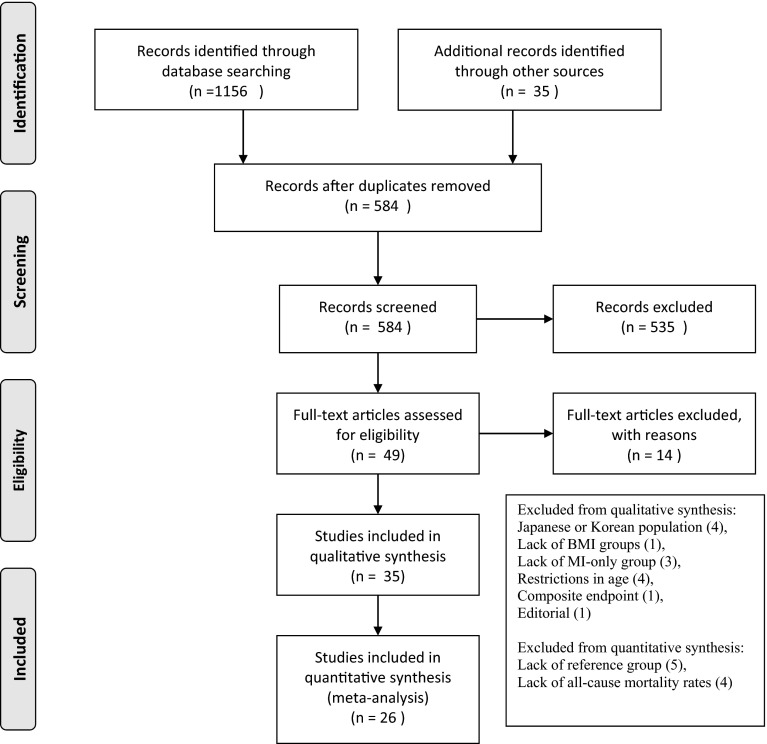
Flow diagram of the study (according to PRISMA statement)

Selection process was shown on Fig. 1 and had been performed according to PRISMA statement [16].

### Study appraisal

Studies included in meta-analysis were appraised independently using Newcastle-Ottawa Quality Assessment Scale. Due to restricted inclusion/exclusion criteria, all of the studies had high (at least **) ratings in adequacy of selection and outcomes assessment. Comparability differed between studies, but meta-analysis was conducted on the basis of unadjusted mortality rates (see “Methodology”). Agreement for the quality of the studies was over 90 %.

### Data extraction

Two reviewers (J.N. and B.H.) screened independently the titles and abstracts for relevance. Discrepancies between reviewers were discussed until consensus was reached. The articles of selected titles/abstracts were reviewed for inclusion. Using the above-mentioned selection criteria, these 2 reviewers determined independently the articles which were included and excluded. The data from the relevant articles were extracted using predefined extraction forms (Supplemental Appendix Table 1, available online). Any disagreements in data extraction were discussed until consensus was reached.

### Methodology

Due to differences in BMI groups between studies in our analysis (see the footnote of Table 2), patients were qualified to the closest BMI group. For the purpose of our meta-analysis subjects were divided into 5 groups: Low BMI, Normal BMI, Overweight, Obesity and Severe obesity. Due to heterogeneity of definitions of underweight used in different studies, in our Low BMI category we included subgroups of patients with BMI below 20 kg/m^2^. Again, Normal BMI was defined as a BMI range from 18.5 to 25 kg/m^2^, because in studies various BMI intervals were used i.e. 20–25 or 18.5–24.9 kg/m^2^ (Table 2). Patients with BMI 25–30 or 30–35 kg/m^2^ were categorized as Overweight and Obesity, respectively. Severe obesity category comprised patients with BMI ≥ 35 kg/m^2^. Patients with BMI 35–39.9 kg/m^2^ and patients with BMI 40 kg/m^2^ or more were pooled as Severe obese (≥35).

**Table 2:** The summary of studies included into meta-analysis

Author	Year	Location	Enrolment period	ACS type	Number of patients	Treatment	Men %	BMI category	Follow up (months)	Prevalence (%)				
										Low BMI	Normal BMI	Overweight	Obesity	Severe obesity
Hoit [17]	1987	USA	1979–1983	AMI	1,760	M	75.4	I	IH + 12	–	37.4	50.2	12.4	–
Lopez-Jimenez [18]	2004	USA	1979–1998	AMI	2,263	M P T	57.7	G	68.4	–	36.0	40.0	24.0	–
Rana [19]	2004	USA	1989–1994	AMI	1,898	NA	69.4	A	45.6	–	32	44	17	7
Eisenstein [20]	2005	International	1997–1999	ACS	15,071	M P T C	72.7	E	12	–	27.0	44.5	20.4	8.1
Kragelund [21]	2005	Denmark	1990–1992	AMI	6,168	M T	67.4	M	96	2.6	42.2	42.3	12.9	–
Diercks [22]	2006	USA	2001–2003	UA/NSTEMI	80,845	M P C	60.4	D	IH	2.9	26.6	35.9	20.8	18.8
Goldberg [23]	2006	USA	1997, 1999, 2001, 2003	AMI	3,513	P C	57.2	F	IH	7.0*	38.5	29.1	15.5	9.9
Iakobishvili [24]	2006	Israel	2002–2003	STEMI	164	P	75.6	J	1.0	–	36.0	42.1	21.9	–
Nikolsky [25]	2006	International	1997–1999	AMI	2,035	P	73.1	G	12	–	27	45	28	–
Wells [26]	2006	USA	2003–2004	AMI	284	M P T C	68.3	L	IH	6.0	22.2	34.2 1	22.9	14.8
Buettner [27]	2007	Germany	1996–1999	UA/NSTEMI	1,676	P	66.0	A	17	0.5*	32.9	49.2	14.6	17.4*
Mehta [28]	2007	International	1990–1997	AMI	2,325	P T	73.9	G	IH	–	30.2	44.7	25.1	–
Lopez-Jimenez [29]	2008	USA	1996–2001	AMI	1,676	M P C	55.9	K	29	3.6	22.8	37.6	30.2	5.8*
Mehta [30]	2008	Germany	1994–2002	STEMI	7,630	P T	70.7	G	IH	–	29.8	49.3	20.8	–
Wienbergen [31]	2008	Germany	1998–2002	STEMI	10,534	M P T C	70.2	D	IH + 14	–	32.3	43.5	20.2	–
Aronson [32]	2010	Israel	2001–2007	AMI	2,157	M P	78.7	B	26	1.2	28.7	44.2	20.1	5.8
Hadi [33]	2010	Middle East	2006–2007	ACS	7,843	P T	75.8	G	IH	–	32.8	40.4	26.7	–
Mahaffey [34]	2010	International	2001–2003	UA/NSTEMI	9,873	M P C	66.2	L	1.0	2.4	23.8	41.5	21.7	10.1
Shechter [35]	2010	Israel	2002, 2004, 2006	ACS	5,751	M P C	77.0	E	12	0.8	29.7	46.9	22.6	–
Das [36]	2011	USA	2007–2009	STEMI	49,329	P T	70.5	D	IH	–	23.5	38.7	22.4	13.8
Timoteo [37]	2011	Portugal	2005–2008	STEMI	539	P	77.0	C	12	–	34.9	46.2	18.9	–
Bucholz [38]	2012	USA	2003–2008	AMI	6,359	M P C	67.4	A	12	–	22.8	36.4	24.1	16.7
Camprubi [39]	2012	Spain	2009–2010	ACS	824	P	73.5	C	IH	–	27.6	50.6	21.8	–
Lazzeri [40]	2012	Italy	2004–2010	STEMI	1,268	P	73.2	O	IH + 12	2.9	31.8	51.7	13.6	–
Herrmann [41]	2014	International	2005–2007	STEMI	3,579	M P C	76.6	H	36	–	29.5	64.3	6.2	–
Witassek [42]	2014	Switzerland	2006–2012	STEMI	6,938	P	77.1	A	IH	1.0	33.1	45.0	15.9	5.0
26 Studies			1979–2012		218,532									

*ACS* acute coronary syndrome, *AMI* acute myocardial infarction, *UA* unstable angina, *NSTEMI* non-ST-elevation myocardial infarction, *STEMI* ST-elevation myocardial infarction, *NA* not applicable/not available, *IH* in-hospital; *USA* United States of America, * No mortality rates/survival analysis for this BMI subgroup (only prevalence available)

Treatment: *M* medical treatment, *T* thrombolysis, *P* percutaneous revascularization, *C* coronary artery bypass surgery (CABG)

Reported BMI categories (kg/m^2^): A—Underweight: <18.5; Normal: 18.5–24.9; Overweight: 25–29.9; Obese: 30–34.9; Severe obese: ≥35; B—Underweight: <18.5; Normal: 18.5–21 AND 21–23.5 (reference) AND 23.5–25; Overweight: 25–26.5 AND 26.5–28 (overweight referent) AND 28–30; Obese: 30–35; Severe obese: ≥35; C—Normal: <25; Overweight: 25–29.9; Obese: >30; D—Underweight <18.5; Normal: 18.5–24.9; Overweight: 25–29.9; Obese (class I) 30–34.9; Obese: (class II) 35–39.9; Obese: (class III) ≥40 (severe obesity = class II + III obesity); E—Underweight: <18.5; Normal: 18.5–24.9; Overweight: 25–29.9; Obese: (class I, II, III) ≥30; F—Normal: <25; Overweight: 25–29.9; Obese: 30–34.9, Severe obese: ≥35; G—Normal: <25; Overweight: 25–29.9; Obese: ≥30; H—Normal: <24.5; Overweight: 24.5–27 AND 27.1–30.1; Obese: >30.1; I—Normal: <25; Overweight: 25–34.9; Obese: >35; J—Normal: ≤25; Overweight: 25–30; Obese: >30; K—Underweight: <20; Normal: 20–24.9; Overweight: 25–29.9; Obese: 30–39.9; Morbidly obese: ≥40 (obesity = ≥30); L—Underweight: <20; Normal: 20–25; Overweight: 25–30; Obese: 30–35; Severe obese: ≥35; M—Underweight: <18.5; Normal: 18.5–24.9; Overweight: 25–29.9; Obese: >30

### Statistical analyses

A random effects model with inverse variance weighting was used to calculate pooled relative risks (RR) and 95 % confidence interval (CI). Total mortality after ACS was analyzed. Unadjusted mortality rates (2 × 2 or risk ratios) in BMI groups were extracted from studies. Normal BMI group was chosen as the reference one. Heterogeneity between studies was assessed using Cochran Q test and I2 statistic, which denotes the percentage of total variation across studies as a result of heterogeneity rather than chance. All heterogeneity results from analyses of each group were compared with those of the Normal-BMI group. Heterogeneity was considered significant if the P value for the heterogeneity test was less than 0.05. Publication bias was tested by using the Begg and Mazumdar rank correlation test and the Egger’s regression intercept test. In case of significant bias, Duval and Tweedie’s trim and fill method was applied to correct the funnel plot asymmetry. The effect of individual studies was examined by exclusion sensitivity analysis. Each study was removed at a time to assess the degree to which the meta-analysis estimate depends on that particular study.

## Results

### Study characteristics

Out of the 49 pre-selected articles, 26 met inclusion criteria for meta-analysis [17–42].

218,532 patients with ACS, enrolled in years 1979–2012 were included in the study. Each study contained more men (range between 55.9 and 78.7 %) than women.

Excluded articles with criterion for exclusion were shown in the frame on Fig. 1. To avoid bias due to the differences in diagnostic criteria of overweight and obesity, data from Japanese and South Korean populations were excluded from the analysis (4 studies).

### Main analysis

**Fig. 2:**
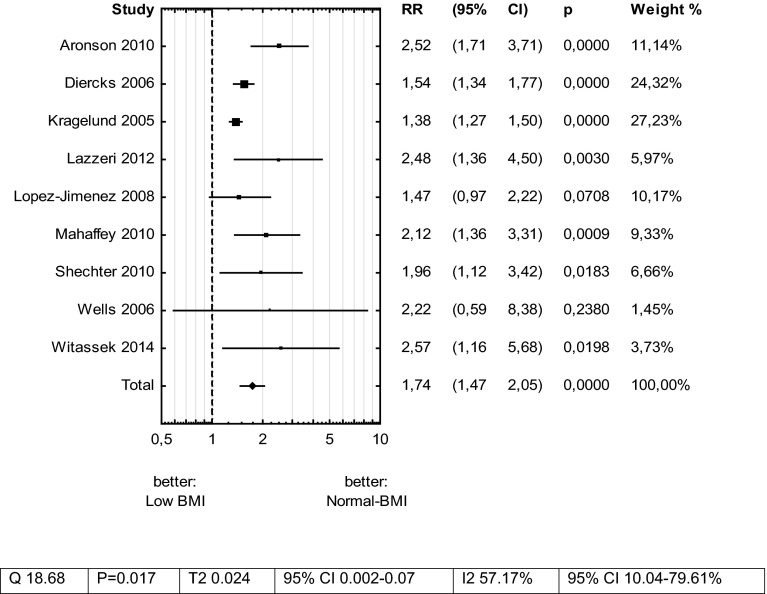
Meta-analysis: total mortality risk for Low BMI versus Normal BMI in patients with acute coronary syndrome

The relative risk ratio for total mortality in patients after ACS with Low BMI was RR 1.74 (CI 1.47–2.05)—Fig. 2. The Begg and Mazumdar rank correlation test was not significant (*p* = 0.47), but Egger’ s regression intercept test showed significant bias for publications (*p* = 0.006). The Duval and Tweedie’s Trim and Fill method was used to impute 5 missing studies and estimate RR as 1.47 (1.24–1.74).

**Fig. 3:**
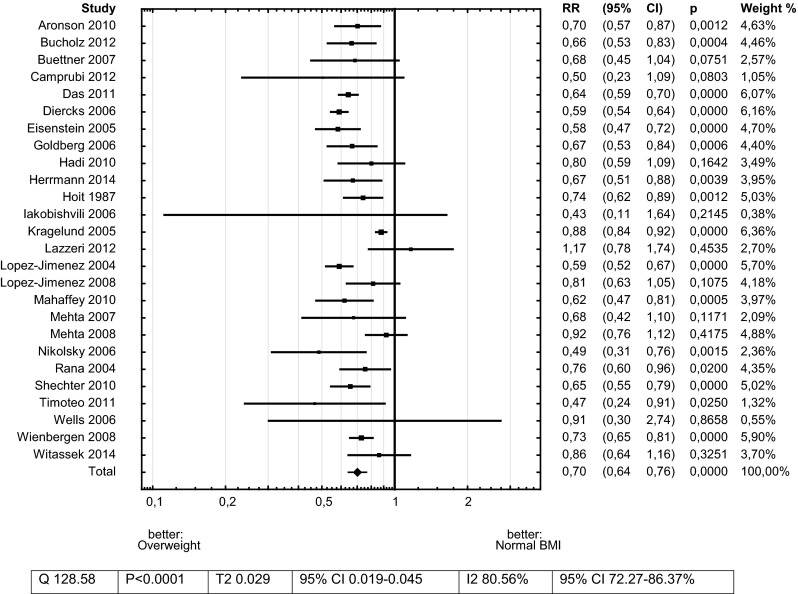
Meta-analysis: total mortality risk for Overweight versus Normal BMI in patients with acute coronary syndrome

Overweight patients had 30 % lower mortality risk after ACS in comparison to those with Normal BMI–RR 0.70 (CI 0.64–0.76)—Fig. 3.

**Fig. 4:**
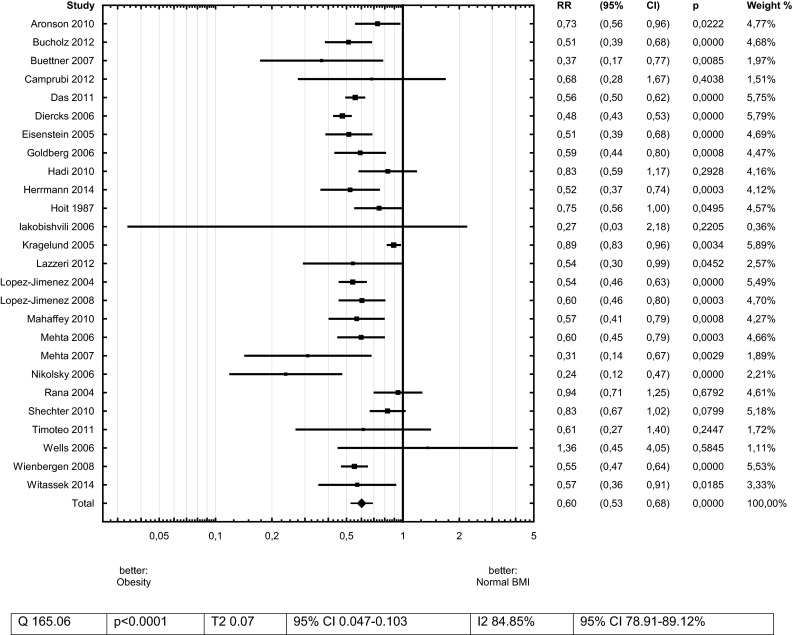
Meta-analysis: total mortality risk for Obesity versus Normal BMI in patients with acute coronary syndrome

Obesity was related to 40 % lower risk of death after ACS in comparison with Normal-BMI subjects—RR 0.60 (95 % CI 0.53–0.68)—Fig. 4.

**Fig. 5:**
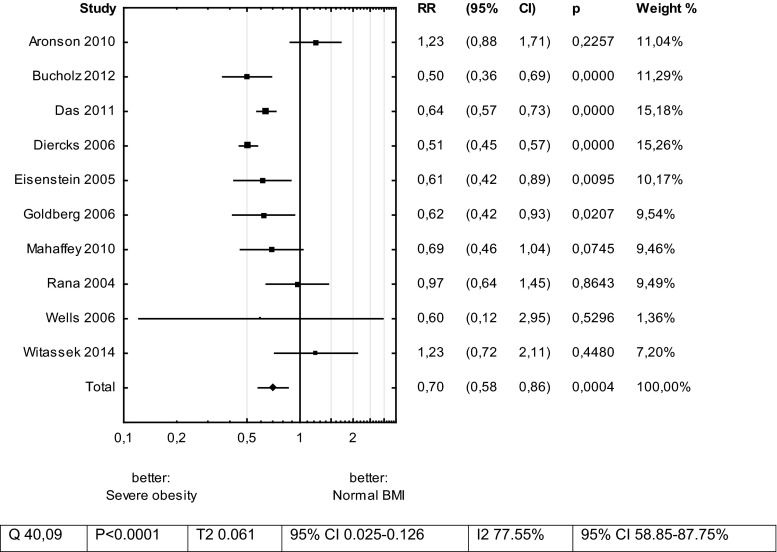
Meta-analysis: total mortality risk for Severe Obesity versus Normal BMI in patients with acute coronary syndrome

Severely obese patients had 30 % lower mortality risk after ACS in comparison to those with Normal BMI—RR 0.70 (CI 0.58–0.86)—Fig. 5.

Both tests used for publication bias assessment were not significant for Overweight, Obesity nor Severe obesity groups.

**Fig. 6:**
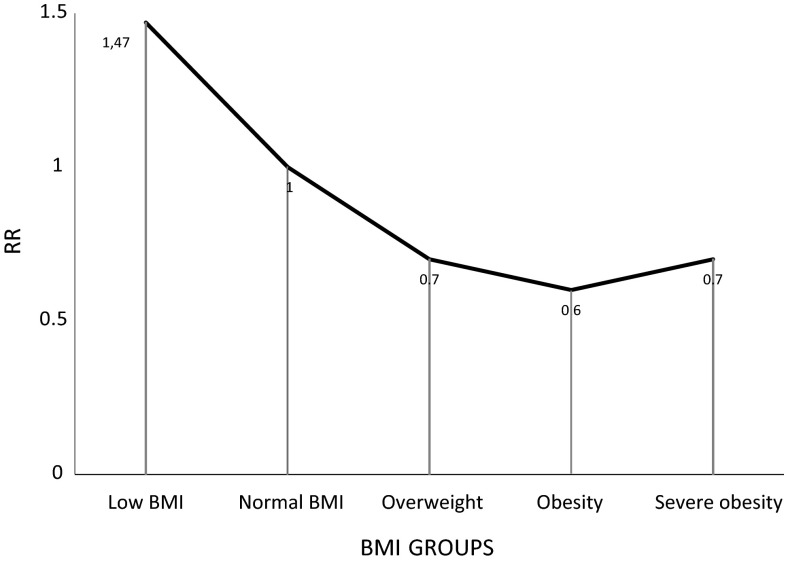
Risk ratios (RR) assessed in meta-analysis in groups of BMI

The relation between risk of mortality and BMI groups was U-shaped—Fig. 6.

## Discussion

### Age and sex

In 20 of 26 studies, overweight and/or obese patients were younger (1–10 years). Madala et al. [43] observed that the first NSTEMI occurred 12 years earlier in severely obese than in normal BMI patients, whilst only 3.5 years earlier in less endangered overweight group. The finding of younger age of obese patients admitted for ACS therapy could be one of possible explanation for the better survival after ACS in people with BMI ≥ 25 kg/m^2^. Peto et al. [44] showed that in general population patients with BMIs above 25 kg/m^2^ had an expected lifetime about 10 years shorter than people with normal BMI. Thus, the percentage of obese people in the population decreases with increasing age.

In patients aged 65 years or older, mortality was higher among obese patients in comparison with those with overweight (*p* < 0.01) and normal weights (*p* < 0.001). Obesity in this age group was an independent risk factor for in-hospital mortality [17].

There are different reports on sex distribution across BMI groups. In some studies (Aronson, Eisenstein) more women, while in others [18, 28, 30] more men were included in the obese groups. Rana et al. [19] showed more women in normal-weight and class 1 and 2 obesity with nadir in the overweight ones (39, 33, 40 and 22 %, respectively, *p* < 0.001). Similar differences were found for cardiogenic shock with occurrence 9.0; 4.1; 3.1; 2.9 and 5.4 % for underweight, normal weight, overweight, class 1 and class 2/3 obesity (*p* = 0.006), respectively [42].

### Comorbidities and complications

Patients with BMI ≥ 25 kg/m^2^ had higher cardiovascular risk. Diabetes mellitus (20 studies), hypertension (20 studies) or hyperlipidemia (10 studies) were more prevalent in obese than in normal-BMI group. Nevertheless, two studies showed lower GRACE risk score in obese patients [35, 38].

Better survival in overweight or obese patients might be due to the relatively short follow-ups in the studies. During in-hospital stay or even in 5 years after MI, diabetes mellitus or hypertension had little chance to evoke complications and impact the mortality.

Although overweight or obese patients smoked rarely [19–21, 28, 33, 35, 41], mortality risk among current smokers was higher in these groups and rose with increasing BMI–hazard ratio (HR) for BMI > 35 kg/m^2^ was 4.51 (95 % CI, 1.42–14.3) in comparison to HR 1.18 (95 % CI, 0.42–2.58) for former smokers [19]. Only 8 % of underweight patients smoked in the past in comparison to 15, 16 and 17 % found in normal-weight, overweight and obese subjects respectively (*p* = 0.001) [21].

Obese patients had higher concentrations of C-reactive protein [27], lower troponin and NT-proBNP levels [45]. The finding of lower natriuretic peptides levels in obese heart failure patients has been recognized recently was and could be explained by clearance function of adipose tissue on these peptides [46].

Compared to normal-BMI group, in obese patients higher estimated glomerular filtration rates by both, MDRD or Cockroft-Gault formulas were observed [25, 36, 47]. The choice of renal function estimation may be important because in patients with coronary artery disease and serum creatinine within normal range, CKD-EPI formula (Chronic Kidney Diseases Epidemiology Initiative) which was derived based on populations with vaster distribution of BMI, predicted long-term outcome more accurately, than MDRD equation [48].

Patients with BMI < 25 had higher risk of bleeding [25, 34]. Nikolsky et al. [25] postulated that the difference had been determined by gastro-intestinal bleeding (2.7 vs 0.4, *p* = 0.02 for normal weight and obesity, respectively). Moreover, overweight and obese more often had anemia [41] and indication for blood transfusion [25]. Noteworthy, the local groin bleeds (hematoma in the arterial puncture site) occurred also more frequently in patients with normal body weight, compared with overweight and obese (11, 6.8 and 7.6 %, respectively, *p* = 0.014) [28]. This phenomenon could be explained by ability of fat tissue to compress punctured femoral artery and staunch bleeding.

Obese patients had less often history of stroke [18, 21] and rarely in-hospital stroke [39], but this also could be explained by the differences in age.

Kragelund et al. [21] showed that prevalence of cancer was more likely in underweight women group: 12 vs 5 %, 3 and 4 % in normal-weight, overweight and obese groups respectively (*p* = 0.001). The observation was confirmed by Angerås et al. [49] (from 8.7 % in underweight to 1.9 % in patients with BMI ≥ 35 kg/m^2^, *p* < 0.001).

### Diagnosis and treatment

Angiotensin converting enzyme inhibitors (ACEI) were used more frequently in obese as compared to normal weight patients with ACS in 9 studies. Similarly beta-blockers (BB) or statins were given with higher probability to obese patients in 12 and 11 studies respectively. Better pharmacological treatment in obese patients might be caused by existence of other indications for these drugs such as hypertension (20 studies) among obese.

In four studies coronary angiography was reported more often in obese patients [22, 23, 33, 34]. Additionally, six studies reported less frequent percutaneous coronary revascularization in underweight or normal-weight patients with ACS [20, 22, 23, 31, 32, 34].

The door-to-balloon time was significantly longer in obese compared with normal weight patients. Moreover, they had more often final TIMI flow grade 0 compared to normal-weight individuals (2.0 vs. 0.4 %, respectively; *p* = 0.04) [28]. Initial TIMI flow grade 0 or 1 was also differs between in normal-weight and overweight patients (1.8 vs 0.7 %, respectively, *p* = 0.04), as well as between overweight and obese subjects (0.7 vs 2.1 %, respectively, *p* = 0.01) [25].

Multi-vessel coronary artery disease was more common in patients with a normal body weight than in obese with BMI ≥ 40 kg/m^2^, according to studies of Das et al. (28.4 vs 22.4 %) and Diercks et al. (30.0 vs 24.6 %) [22, 36]. Nikolsky et al. [25] did not confirm the higher occurrence of multi-vessel coronary artery disease in normal-weight with STEMI and showed the same frequency of percutaneous (and surgical) revascularization in all BMI ranges.

Despite the lack of differences in the effect of angioplasty, patients with normal weight required a longer hospital stay: 7.1, 6.9, and 6.7 days for normal weight, overweight, and obese, respectively; *p* = 0.014. Major adverse cardiovascular events (MACE) at 6 months was also observed more often in the normal BMI range in comparison with overweight and obese cases: 8.8, 6.6, and 5.0 % respectively; *p* = 0.031 [28]. Major adverse cardiovascular or cerebrovascular events (MACCE) was also more frequent in normal-weight patients, comparing to overweight and obese subjects: 14.7, 12.7, 10.0 %, respectively for in-hospital outcome (*p* < 0.001) and 12.6, 9.3, 8.7 %, respectively (*p* < 0.001) for long-term follow-up [31].

### Central obesity and weight loss

Only four studies highlighted the prognostic role of central obesity. Zeller et al. divided patients with myocardial infarction (MI) into the tertiles of BMI and waist circumference (WC). The group of lower or middle tertile of BMI and upper tertile of WC had 1-year mortality risk above 20 % in women and more than 18 % in men, whilst in lower WC and upper BMI tertiles mortality was 7.6 and 7.7 %, respectively [50]. This finding was confirmed by Kadakia et al. [45]. It may indicate the special significance of central obesity. Unfortunately, most of the studies did not report parameters allowing more detailed description of obesity phenotype. Kragelund et al. [21] confirmed abdominal obesity assessed by waist-to-hip ratio, to be independent predictor of all-cause mortality in men (adjusted RR 1.22 (1.07–1.38), *p* < 0.01), but not in women subgroup after ACS [adjusted RR 1.13 (0.95–1.34, *p* = 0.2)].

Guidelines of European Society of Cardiology (ESC) for the prevention of cardiovascular disease in clinical practice, highlights that obesity in the general population is associated with an increased incidence of cardiovascular disease and cardiovascular mortality. Therefore, the recommendation (class I, level of evidence A) exists for a weight reduction of overweight or obese individuals who have not undergone any cardiovascular event. Body weight reduction to the normal range (BMI 20–24.9 a kg/m^2^) has a positive effect on blood pressure and plasma lipids, which is reflected in a lower incidence of cardiovascular disease [51]. So far, no studies have confirmed the mortality reduction after MI in patients who reduced their body weight [52]. On the contrary, weight loss of more than 5 % after MI in patients with depression (found in 27 % of patients) was related to 70 % higher risk of all-cause and cardiovascular mortality and those finding were not associated with depression nor social support [29]. Weight loss of more than 5 % in a South Korean population of patients following acute MI was associated with a higher 1-year rate of MACEs. Patients who gained weight also have a greater 1-year mortality risk [7]. On the other hand, intentional weight loss during cardiac rehabilitation in patients with CAD (not MI) was a marker for favourable long-term (6.4 years) outcomes, in both subgroups with initial BMI < 25 or ≥25 kg/m^2^ [53].

### Comparison to general population

The collected data showed that in a population of patients with ACS, an obesity paradox may occur. However, a meta-analysis of 97 studies about mortality in the general population, published in January 2013, indirectly calls into question the existence of the obesity paradox in patients with ACS and chronic diseases. In the general population, the risk of death (HR) in people who were overweight and in the 1st class of obesity (BMI 25–35 kg/m^2^) was lower than in individuals with normal weights (BMI 18.5–25 kg/m^2^). Only patients with BMIs 35 kg/m^2^ and greater had a higher risk of death [54]. To compare the results of the studies about BMI and mortality in chronic diseases with the work of Flegal et al. [54], the obesity paradox exists also in the general population. In the ACS, chronic diseases and the general population the lowest mortality was observed among individuals with BMI values above the normal WHO range.

Although results of our study seem to be clear and quite obvious, outcomes should be interpreted with caution. Despite obese patients more often had diabetes mellitus and/or hypertension, they were younger and had less bleeding complications. Therefore, to compare the mortality of obese patients with people with normal BMIs, the age of the patients and associated diseases should be taken into account in long enough follow-up. In other cases, the relationship between BMI and mortality may be disturbed.

In unadjusted analyses performed on data assessed from the studies, better survival in overweight, obesity and severe obesity group was confirmed in 16 out of 26 studies, 19 of 26 and 5 of 10 studies, respectively. In Low BMI group 7 of 9 studies showed worse survival, comparing to Normal BMI group. After adjustment, both for multivariate analysis (BMI as continuous variable) or models adjusted for various covariables (BMI groups), significant relation between lower BMI and worse survival was found in 15 out of 25 studies.

## Conclusion

The existence of obesity paradox in patients with ACS is supported by our meta-analysis.

### Limitations

Our study has some limitations and weaknesses.

The analyzed articles varied in methodology. Groups of BMI were categorized using 11 different classification (see footnote of Table 2). Thus, in some studies BMI 19 kg/m^2^ was classified as ‘Low BMI’, in other—as ‘Normal BMI’. In some publications, underweight patients were excluded from the analyses, because of the ‘extreme high risk of mortality’ [38].

There were lacks of detailed data on race, age, treatment or complications in most of studies, thus those parameters were not shown in the analysis.

The reliability of the data on height and weight is also an important issue. Significant discrepancies between the values measured by physicians and those reported by patients have been shown [54]. Nevertheless, in most ACS cases, weight and height measurements are not possible to conduct, due to life-threatening condition.

## Electronic supplementary material

Below is the link to the electronic supplementary material.

Supplementary material 1 (DOCX 12 kb)
