# Supplementary material for: The obesity paradox in acute coronary syndrome: a meta-analysis
Source: Eur J Epidemiol. 2014 Oct 30;29(11):801–12. doi: 10.1007/s10654-014-9961-9 (PMC4220102; doi:10.1007/s10654-014-9961-9)
Supplement: Supplementary file 1 — Supplementary material 1 (DOCX 12 kb) [file 10654_2014_9961_MOESM1_ESM.docx]

Supplemental Appendix Table 1. Search strategy

| 1. Define your question using PICOS | Table 1 |
| --- | --- |
| 2. Type of question/problem | Prognosis |
| 3. Type of study (Publication Type) to include in the search | Randomized controlled trials, Non-randomized controlled trials, Retrospective, prospective, or concurrent cohort studies, Cross sectional studies, Case Control Study |
| 4. List main topics and alternate terms from your PICOS question that can be used for your search | - body mass (‘body mass index’ or BMI or ‘body weight’ or obesity or overweight or underweight)  - patient/problem (‘acute coronary syndrome’ or ‘myocardial infarction’ or ‘unstable angina’)  - outcome (mortality or death) |
| List your inclusion criteria – sex, age, year of publication, language | Sex: men and women; Age: adults; Year of publication: all; Language: English; Human studies, |
| List irrelevant terms that you may want to exclude in your search |  |
| List where you plan to search | PubMed, Cochrane Library, ScienceDirect |

PICOS - patients, intervention, comparator, outcomes, study design; ACS – acute coronary syndrome; BMI – body mass index;
